# Zingiberensis Newsaponin Inhibits the Malignant Progression of Hepatocellular Carcinoma via Suppressing Autophagy Moderated by the AKR1C1-Mediated JAK2/STAT3 Pathway

**DOI:** 10.1155/2021/4055209

**Published:** 2021-12-13

**Authors:** Keqing He, Xing Liu, Shiping Cheng, Pingsheng Zhou

**Affiliations:** ^1^Department of Hepatobiliary Diseases, Affiliated Hospital of Jiangxi University of Chinese Medicine, Nanchang, Jiangxi Province 330006, China; ^2^Department of Medicine, Jinggangshan University, Qingyuan District, Jian, Jiangxi Province 343009, China; ^3^Department of Dermatology, Affiliated Hospital of Jiangxi University of Chinese Medicine, Nanchang, Jiangxi Province 330006, China; ^4^Jiangxi University of Chinese Medicine, Nanchang, Jiangxi Province 330006, China

## Abstract

**Objective:**

Saponins are a group of compounds from various plants, which exhibit an anticancer activity. This study aimed to explore the anticancer effect of zingiberensis newsaponin (ZnS) against hepatocellular carcinoma (HCC) and the underlying mechanism involving autophagy.

**Methods:**

HCC cells (Huh7 and SMMC7721) were treated with ZnS and/or 3-MA. The cell viability, migration, and apoptosis were determined using CCK-8 assay, transwell assay, and flow cytometry, respectively. The levels of oxidative stress markers (ROS, SOD, and MDA) were measured by ELISA assay. Autophagy was monitored using MDC assay, immunofluorescence staining, and transmission electron microscopy. The relative protein expression of LC3II/LC3I, P62, AKR1C1, p-JAK2, p-STAT3, JAK2, and STAT3 was determined using Western blot.

**Results:**

ZnS or 3-MA inhibited the cell viability and migration, and it promoted cell apoptosis and oxidative stress in HCC. MDC-positive cells and autophagosomes were reduced by ZnS or 3-MA treatment. The expression of autophagy-related proteins LC3 (LC3II/LC3I) and P62 was, respectively, downregulated and upregulated after ZnS or 3-MA treatment. In addition, ZnS or 3-MA suppressed the protein expression of AKR1C1, p-JAK2, and p-STAT3 in HCC cells. Furthermore, the above phenomena were evidently enhanced by ZnS combined 3-MA treatment. AKR1C1 overexpression weakened the effect of ZnS on inhibiting the expression of AKR1C1, p-JAK2, and p-STAT3.

**Conclusion:**

ZnS exerts an anticancer effect on HCC via inhibiting autophagy moderated by the AKR1C1-mediated JAK2/STAT3 pathway. ZnS and 3-MA exert a synergistic effect on inhibiting HCC.

## 1. Introduction

Hepatocellular carcinoma (HCC) is considered as a leading cancer across the globe, which is closely related to various liver diseases [1]. The incidence and mortality of HCC are rapidly increasing worldwide, and there are millions of new cases found each year [2]. Currently, despite numerous advanced therapies and technologies applied for HCC treatment, the therapeutic outcomes are still unsatisfactory [3]. Hence, it is of importance to explore more efficacious and tolerable drugs for HCC therapy.

Saponin is a class of secondary metabolite from diverse plants, including Dioscoreaceae, Liliaceae, and Scrophulariaceae. Saponin has been widely applied for the treatment of various cancers, such as breast cancer, colon cancer, and liver cancer, due to its favorable physiological and pharmacological activities, including immunomodulation, antioxidation, and antiapoptosis [4–6]. Zingiberensis newsaponin (ZnS) is the main bioactive ingredient in the rhizome of *Dioscorea zingiberensis* C.H. Wright, which has been applied for the treatment of cough, rheumatic heart disease, and coronary heart disease for many years in China [7]. Previous literatures have confirmed that ZnS is the potential drug against cancers, such as colon cancer and gastric cancer, with high efficiency and low toxicity [8–11]. However, the underlying mechanism of ZnS against HCC remains largely elusive.

Autophagy is a highly conserved and tightly orchestrated cellular process for the degradation of sequesters misfolded proteins, damaged or aged organelles, and mutated proteins, which is critical for maintaining homeostasis under stress conditions [12]. Dysregulated autophagy results in a series of negative implications in health and disease, such as cardiomyopathy, infectious disease, fatty liver, and cancer [13–15]. Autophagy has been identified as a target for drug therapeutic intervention in cancers. For instance, xanthoangelol exerted therapeutic effects on human hepatocellular carcinoma through inducing protective autophagy [16]. Soyasapogenol B could suppress colorectal cancer via inducing apoptosis and autophagy [17]. However, it remains unclear how ZnS inhibits HCC by regulating autophagy.

Aldo-keto reductase 1C1 (AKR1C1) is a member of the human aldo-keto reductase family, which is responsible for the catalyzation of NADPH-dependent reductions [18]. Accumulating evidence indicates that AKR1C1 is an essential regulator in various cancers, including liver cancer, prostate cancer, and breast cancer [19, 20]. In addition, AKR1C1 is a pivotal component of the Janus kinase 2/signal transducer and activator of transcription 3 (JAK2/STAT3) pathway [18, 21]. The JAK2/STAT3 signaling pathway acts as a crucial role in regulating the anticancer immune response, which has emerged as a promising therapeutic strategy for cancers [22, 23]. Furthermore, growing studies have confirmed that the JAK2/STAT3 pathway exerts the anticancer effect via the regulation of autophagy [24–26]. However, whether ZnS exerts an inhibitory effect on HCC via impacting autophagy moderated by the AKR1C1-mediated JAK2/STAT3 signaling pathway remains unclear.

To this background, this research investigated the inhibitory effect of ZnS on HCC by evaluating cell viability, migration, apoptosis, and oxidative stress. Simultaneously, the underlying mechanisms of ZnS involving autophagy and AKR1C1-mediated JAK2/STAT3 signaling pathway were unveiled. These results throw lights into the underlying mechanism of ZnS against cancer and provide a potential drug for HCC therapy.

## 2. Materials and Methods

### 2.1. Cell Culture and Treatment

Human HCC cells (Huh7 and SMMC7721) were purchased from the Cell Bank, Shanghai Institutes for Biological Sciences, Chinese Academy of Science (Shanghai, China). HCC cells were routinely cultured in Dulbecco's modified Eagle's medium (DMEM) containing 1% penicillin/streptomycin and 10% fetal bovine serum (FBS) (HyClone, UT, USA) with an atmosphere of 5% CO_2_ at 37°C. A part of cells were treated with different concentrations of ZnS (0.1, 0.2, 0.4, 0.8, and 1.6 *μ*M). Other cells were divided into four groups: the control group (no treatment); the 3-methyladenine (3-MA) group, in which cells were pretreated with 10 mM 3-MA (an autophagy inhibitor) for 6 h; the ZnS group, in which Huh7 cells were treated with 0.5 *μ*M ZnS and SMMC7721 cells were treated with 1.0 *μ*M ZnS; and the ZnS + 3-MA group, in which cells were pretreated with 10 mM 3-MA for 6 h, then Huh7 cells were treated with 0.5 *μ*M ZnS, and SMMC7721 cells were treated with 1.0 *μ*M ZnS. After treatment for 48 h, cells were utilized for follow-up experiments.

### 2.2. Cell Counting Kit-8 (CCK-8) Assay

The cell proliferation and viability of Huh7 and SMMC7721 were evaluated by CCK-8 assay following the manufacturer's instruction. The suspended cells (5 × 10^5^ cells/mL) were seeded into 96-well plates (100 *μ*L/well) for incubating at 37°C and 5% CO_2_. Cells cultured for 24 h, 48 h, and 72 h were, respectively, incubated with 10 *μ*L CCK-8 solution (Beyotime, China) for 2 h. The absorbance was measured at a wavelength of 450 nm using a DR-200 Bs microplate reader (Diatek, China). In addition, the relative half-maximal inhibitory concentration (IC50) value of ZnS was identified at 48 h postculturing based on the cell proliferation curve.

### 2.3. Transwell Migration Assay

The migration ability of Huh7 and SMMC7721 cells was evaluated using a transwell assay. Matrigel was coated on the upper chamber of the transwell, and cells were suspended with serum-free DMEM to 5 × 10^5^ cells/mL. Then, 100 *μ*L cells were added to the upper chamber of the transwell; meantime, 500 *μ*L DMEM containing 10% FBS was added to the lower chamber for incubating in an atmosphere of 5% CO_2_ at 37°C for 24 h. Next, cells in the lower chamber were fixed in the pro-cool methanol for 30 min, followed by staining with hematoxylin for 1 min. The stained cells were observed under an optical microscope (400x magnification, Olympus, Japan) through selecting six fields randomly.

### 2.4. Flow Cytometry Assay

The apoptosis levels of Huh7 and SMMC7721 cells were measured following the instructions of the Annexin V-FITC apoptosis kits (Beyotime, China). The suspended cells (5 × 10^5^ cells/mL, 100 *μ*L) were incubated with 5 *μ*L Annexin V-FITC for 15 min at room temperature in the dark, followed by the incubation with 10 *μ*L propidium iodide (PI) for 10 min. Subsequently, the apoptosis was detected using a flow cytometry (Beckman Coulter, Germany). The apoptosis ratios were analyzed using a CellQuest software (BD Biosciences, NJ, USA).

### 2.5. Enzyme-Linked Immunosorbent Assay (ELISA)

For assessment of oxidative stress of Huh7 and SMMC7721 cells, the levels of oxidative stress markers, including reactive oxygen species (ROS), superoxide dismutase (SOD), and malondialdehyde (MDA), were determined using corresponding ELISA kits (Beyotime, China) according to manufacturer's instruction.

### 2.6. Monodansylcadaverine (MDC) Staining

In order to investigate the autophagy in Huh7 and SMMC7721 cells, a MDC kit (Solarbio, China) was utilized according to the manufacturer's instruction. Briefly, 90 *µ*L resuspended cells (1 × 10^6^ cells/mL) were stained with 10 *µ*L MDC solution for 45 min at room temperature in the dark. Images were captured using a confocal microscope (Olympus, Japan), and MDC positive cells were counted.

### 2.7. Transmission Electron Microscope

In order to observe the autophagosome formation in Huh7 and SMMC7721 cells, cells were fixed with 1% pentylene glycol for 2 h prior to fixing with 1% osmium acid buffer for 1 h. Then, cells were dehydrated through an acetone gradient and stained with uranyl acetate and lead citrate. Autophagosomes in Huh7 and SMMC7721 cells were observed using a transmission electron microscope (Thermo Fisher Scientific, MA, USA).

### 2.8. Immunofluorescence Staining

The immunofluorescence staining was performed to evaluate the expression of LC3 (an autophagy-related protein) in Huh7 and SMMC7721 cells according to the description of Li et al. [27]. Briefly, Huh7 and SMMC7721 cells were fixed with 4% paraformaldehyde for 15 min at 4°C and then permeabilized with 0.2% Triton X-100 in PBS for 15 min at room temperature. Next, cells were incubated with primary antibody LC3 (1 : 200, Abcam, UK) at 4°C overnight, followed by incubating with secondary antibody (1 : 300, MultiSciences, China) for 90 min at room temperature. After washed with PBS, cells were stained with DAPI for nuclear counterstaining. Subsequently, the images of stained cells were captured using a confocal microscope (Olympus, Japan).

### 2.9. Cell Transfection

The overexpression of AKR1C1 in Huh7 and SMMC7721 cells was established using the lentivirus packaging system. Briefly, negative control (lenti-NC) and AKR1C1 (lenti-AKR1C1) were packaged in lentivirus. Huh7 and SMMC7721 cells were seeded in six-well plates (2 × 10^5^ cells/mL/well) for culturing at 37°C with 5% CO_2_. When cell confluence reached 80%, lentivirus solutions (lenti-NC and lenti-AKR1C1 with titration of 1 × 10^8^ TU/mL) were added to the six-well plates (20 *μ*L/well) for transfection for 72 h.

### 2.10. Western Blot

Huh7 and SMMC7721 cells were lysed in a RIPA lysis buffer (Takara Bio, Japan) for the extraction of total protein. The protein concentration was quantified using a BCA Protein Assay Kit (Thermo Fisher Scientific, CA, USA). Total proteins were separated by SDS-PAGE and then transferred onto polyvinylidene difluoride (PVDF) membranes. Membranes were incubated with blocking buffer (5% nonfat dry milk dissolved in 1× Tris buffered saline with 0.1% Tween-20) at room temperature for 1 h, followed by the primary antibodies, including LC3II/LC3I, P62, AKR1C1, p-JAK2, p-STAT3, JAK2, STAT3, and GAPDH (1 : 1000, Abcam, UK), at 4°C overnight. Then, membranes were incubated with horseradish peroxidase (HRP)-conjugated secondary antibody (1 : 500, MultiSciences, China) for 1 h at room temperature in the dark. Protein bands were presented using ECL reagent kit (Thermo Fisher Scientific, CA, USA), and images were captured using a ChemiDoc™ imaging system (Bio-Rad, CA, USA). The relative protein expression was quantified by calculating the band density normalized to GAPDH.

### 2.11. Statistical Analysis

All data were presented as mean ± standard deviation. Statistical analyses were conducted using SPSS 27.0 software (IBM, IL, USA). Statistical significance between different groups was analyzed using one-way of analysis variance (ANOVA), followed by Tukey's test. *P* < 0.05 was considered statistically significant differences.

## 3. Results

### 3.1. ZnS Suppresses the Proliferation of HCC Cells

**Figure 1:**
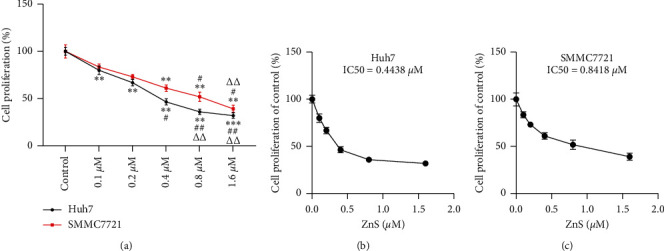
Zingiberensis newsaponin (ZnS) suppresses the proliferation of HCC cells in a dose-dependent manner. (a) The proliferation of HCC cell lines (Huh7 and SMMC7721) measured by Cell Counting Kit-8 (CCK-8) assay. Cells were treated with different concentrations of ZnS (0.1, 0.2, 0.4, 0.8, and 1.6 *µ*M) for 48 h. (b)-(c) The IC50 value of ZnS determined by a proliferation curve at 48 h posttreatment in Huh7 and SMMC7721 cells. ^*∗∗*^*P* < 0.01 and ^*∗∗∗*^*P* < 0.001 vs. the control group. ^#^*P* < 0.05 and ^##^*P* < 0.01 vs. the 0.1 *µ*M ZnS group. ^ΔΔ^*P* < 0.01 vs. the 0.2 *µ*M ZnS group.

To confirm the anticancer efficacy of ZnS on HCC, the proliferation of HCC cell lines (Huh7 and SMMC7721 cells) was evaluated following treatment with different concentrations of ZnS (0.1–1.6 *µ*M) for 48 h. As shown in Figure 1(a), ZnS treatment significantly reduced the proliferation ability of Huh7 and SMMC7721 cells in a dose-dependent manner (*P*< 0.05). The IC50 values of ZnS in Huh7 and SMMC7721 cells were 0.4438 *µ*M and 0.8418 *μ*M, respectively (Figures 1(b) and 1(c)). Therefore, the concentrations of 0.5 *µ*M and 1.0 *µ*M ZnS were, respectively, used to treat Huh7 and SMMC7721 cells in the subsequent experiments.

### 3.2. The Inhibition of Autophagy Enhances the Anticancer Effect of ZnS on HCC Cells

**Figure 2:**
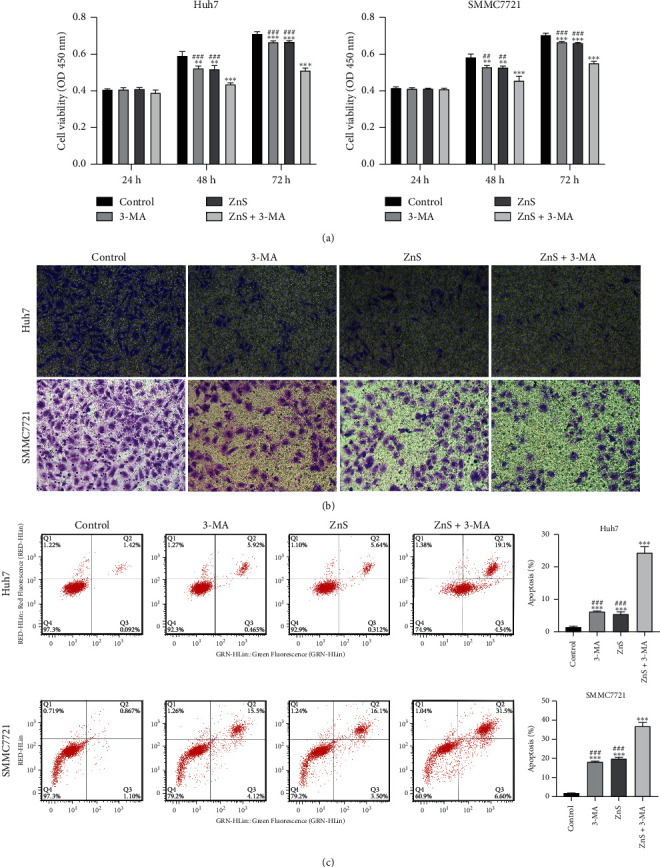
ZnS inhibits the viability and migration, as well as promotes apoptosis of HCC cells. (a) The viability of Huh7 and SMMC7721 cells assessed by CCK-8 assay. (b) The migration ability of Huh7 and SMMC7721 cells examined by transwell assay (400*x* magnification). (c) The apoptosis levels of Huh7 and SMMC7721 cells measured using flow cytometry assay. Cells were treated with 0.5 *µ*M ZnS for Huh7 and 1.0 *µ*M for SMMC7721 cells and/or 10 mM 3-methyladenine (3-MA). ^*∗∗*^*P* < 0.01 and ^*∗∗∗*^*P* < 0.001 vs. the control group. ^##^*P* < 0.01 and ^###^*P* < 0.001 vs. the ZnS + 3-MA group.

In order to investigate the effects of ZnS on HCC, the viability, migration, and apoptosis of Huh7 and SMMC7721 cells were evaluated after ZnS treatment. The results showed that ZnS significantly inhibited the viability of Huh7 and SMMC7721 cells after the treatment for 48 h and 72 h compared with the control group (*P* < 0.01) (Figure 2(a)). The migratory potential of Huh7 and SMMC7721 cells also presented a descending trend after ZnS treatment compared to control cells (Figure 2(b)). Besides, ZnS remarkably promoted the apoptosis of Huh7 and SMMC7721 cells in comparison to the control cells (*P* < 0.001) (Figure 2(c)).

Our previous study has shown that the anticancer activity of ZnS against HCC may be involved with the inhibition of autophagy [28]. Hence, the effects of 3-MA (a specific autophagy inhibitor) on HCC were simultaneously investigated in this study. We found that 3-MA presented the same effects with ZnS, significantly inhibiting the viability and migration, and it promoted the apoptosis of Huh7 and SMMC7721 cells (*P* < 0.01) (Figures 2(a)–2(c)). Furthermore, ZnS combined with 3-MA showed a better anticancer effect on HCC than 3-MA or ZnS alone (*P* < 0.01) (Figures 2(a)–2(c)).

### 3.3. The Inhibition of Autophagy Promotes Oxidative Stress Induced by ZnS in HCC Cells

**Figure 3:**
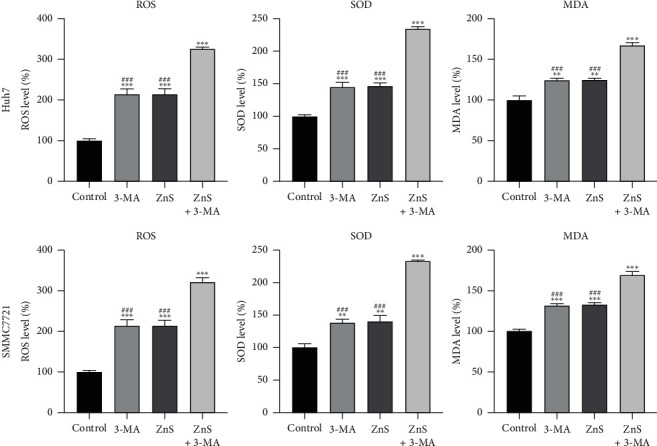
ZnS induces oxidative stress in HCC cells. The levels of reactive oxygen species (ROS), superoxide dismutase (SOD), and malondialdehyde (MDA) in Huh7 and SMMC7721 cells measured by enzyme-linked immunosorbent assay. Cells were treated with 0.5 *µ*M ZnS for Huh7 and 1.0 *µ*M for SMMC7721 cells and/or 10 mM 3-methyladenine (3-MA) for 48 h. ^*∗∗*^*P* < 0.01 and ^*∗∗∗*^*P* < 0.001 vs. the control group. ^###^*P* < 0.001 vs. the ZnS + 3-MA group.

ROS, SOD, and MDA are direct indicators of oxidative stress, which can induce apoptosis and necrosis. 3-MA or ZnS significantly promoted the production of ROS, SOD, and MDA in Huh7 and SMMC7721 cells compared with that in the control cells (*P* < 0.01) (Figure 3). Moreover, ZnS + 3-MA presented a better antioxidative effect on HCC cells than 3-MA or ZnS (*P* < 0.001).

### 3.4. ZnS Suppresses Autophagy in HCC Cells

**Figure 4:**
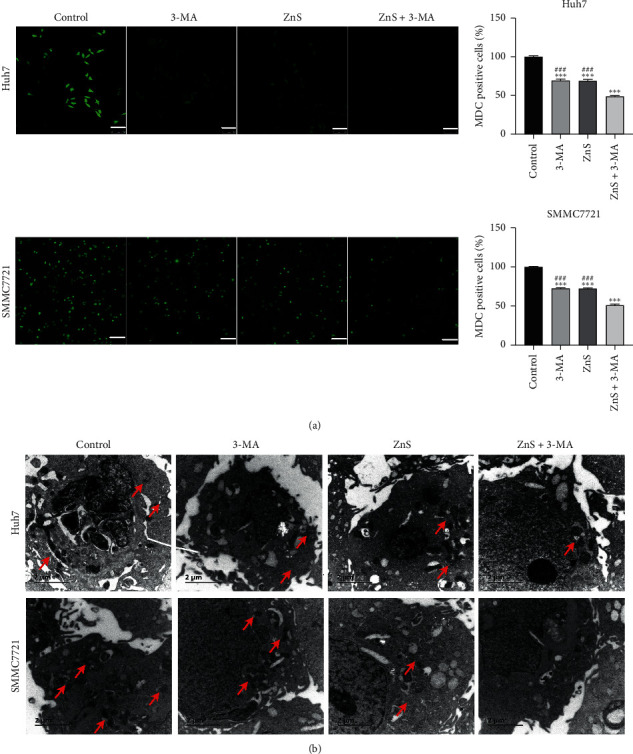
ZnS suppresses autophagosome formation in HCC cells. (a) Monodansylcadaverine (MDC) staining used for the assessment of autophagy in Huh7 and SMMC7721 cells. Bar = 100 *µ*m. (b) Transmission electron microscope utilized for the detection of autophagosomes in Huh7 and SMMC7721 cells. Bar = 2 *µ*m. Cells were treated with 0.5 *µ*M ZnS for Huh7 and 1.0 *µ*M for SMMC7721 cells and/or 10 mM 3-methyladenine (3-MA) for 48 h. ^*∗∗∗*^*P* < 0.001 vs. the control group. ^###^*P* < 0.001 vs. the ZnS + 3-MA group.

MDC is a fluorescent compound commonly applied for labeling autophagosomes in cells. The staining results showed that MDC positive cells significantly decreased after 3-MA or ZnS treatment compared with the control group (*P* < 0.001) (Figure 4(a)). The MDC-positive cells in the ZnS + 3-MA group presented a more obvious reduction in comparison to that in the 3-MA or ZnS group (*P* < 0.001). In addition, the autophagosome formation was observed under a transmission electron microscope. After 3-MA or ZnS treatment, autophagosomes in Huh7 and SMMC7721 cells relatively decreased compared with that in the control cells, and ZnS + 3-MA showed a more obvious reduction (Figure 4(b)).

**Figure 5:**
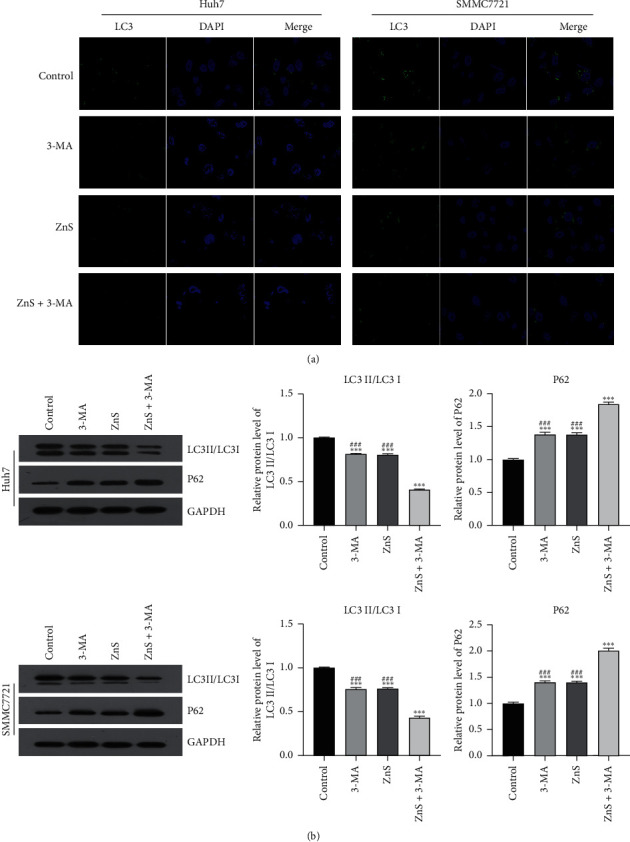
ZnS inhibits the expression of autophagy-related proteins in HCC cells. (a) The expression of LC3 in Huh7 and SMMC7721 cells measured using immunofluorescence staining. (b) The relative expression of LC3II/LC3I and P62 examined by Western blot. Cells were treated with 0.5 *µ*M ZnS for Huh7 and 1.0 *µ*M for SMMC7721 cells and/or 10 mM 3-methyladenine (3-MA) for 48 h. ^*∗∗∗*^*P* < 0.001 vs. the control group. ^###^*P* < 0.001 vs. the ZnS + 3-MA group.

Moreover, the expression of LC3 (an autophagy-related protein) was observed through immunofluorescent staining. The expression level of LC3 in Huh7 and SMMC7721 cells decreased after 3-MA or ZnS treatment compared with that in the control group (Figure 5(a)). Similarly, a lower LC3 expression was observed in Huh7 and SMMC7721 cells after the treatment of ZnS + 3-MA than either ZnS or 3-MA separately. Furthermore, LC3II/LC3I and P62 are important autophagy-related proteins involved in autophagosome formation and maturation. The expression levels of LC3II/LC3I were markedly decreased after 3-MA or ZnS treatment in comparison to the control group; meanwhile, the expression of P62 presented a significant increasing trend in Huh7 and SMMC7721 cells treated with 3-MA or ZnS (*P* < 0.001) (Figure 5(b)). Likewise, ZnS + 3-MA treatment together exhibited a better inhibitory effect on autophagy of Huh7 and SMMC7721 cells than 3-MA or ZnS treatment, evidenced by the decreasing expression of LC3II/LC3I and the increasing expression of P62 (*P* < 0.001).

### 3.5. ZnS Inhibits the AKR1C1-Mediated JAK2/STAT3 Pathway in HCC Cells

**Figure 6:**
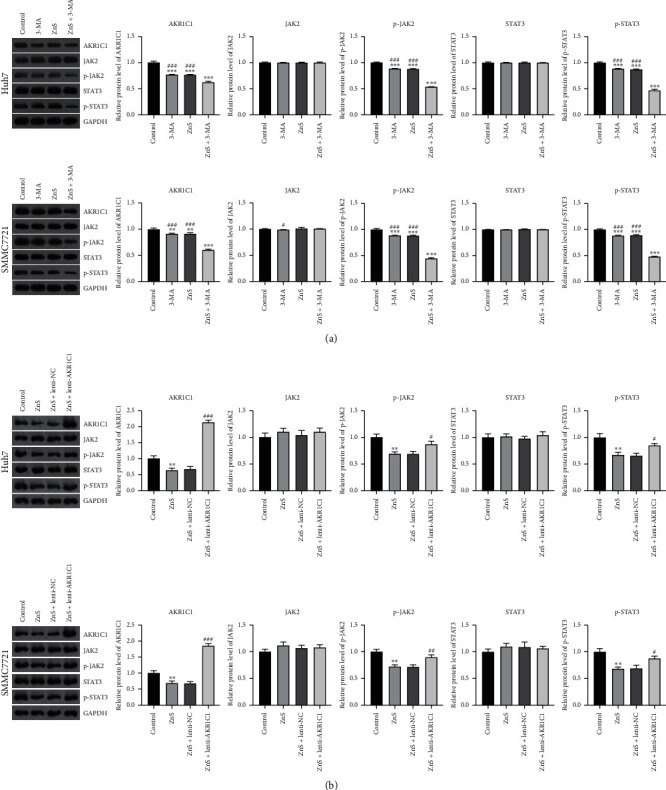
ZnS inhibits the AKR1C1-mediated JAK2/STAT3 pathway. (a) The relative protein expression of AKR1C1, JAK2, p-JAK2, STAT3, and p-STAT3 in Huh7 and SMMC7721 cells examined by Western blot. Cells were treated with 0.5 *µ*M ZnS for Huh7 and 1.0 *µ*M for SMMC7721 cells and/or 10 mM 3-methyladenine (3-MA) for 48 h. ^*∗∗*^*P* < 0.01 and ^*∗∗∗*^*P* < 0.001 vs. the control group. ^#^*P* < 0.05 and ^###^*P* < 0.001 vs. the ZnS + 3-MA group. (b) The relative protein levels of AKR1C1, JAK2, p-JAK2, STAT3, and p-STAT3 in Huh7 and SMMC7721 cells measured by Western blot. Huh7 and SMMC7721 cells treated with ZnS and/or lenti-NC/lenti-AKR1C1. ^*∗∗*^*P* < 0.01 vs. the control group. ^#^*P* < 0.05, ^##^*P* < 0.01, and ^###^*P* < 0.001 vs. the ZnS + lenti-NC group.

AKR1C1 is a member of the human aldo-keto reductase family associated with the occurrence of various cancers. Previous studies confirmed that AKR1C1 promotes tumor metastasis via phosphorylating STAT3 and its upstream kinase JAK2 (JAK2/STAT3 pathway). As shown in Figure 6(a), the expression of AKR1C1, phosphorylated STAT3 (p-STAT3), and phosphorylated JAK2 (p-JAK2) in HCC cells was significantly downregulated after 3-MA or ZnS treatment when compared with that in the control cells (*P* < 0.01). ZnS + 3-MA presented a more commendable effect on downregulating the expression of AKR1C1, p-JAK2, and p-STAT3 in comparison to 3-MA or ZnS (*P* < 0.001). However, the expression of JAK2 and STAT3 did not show significant differences among different groups (Figure 6(a)). In addition, AKR1C1 overexpression dramatically weakened the inhibitory effects of ZnS on the expression of AKR1C1, p-JAK2, and p-STAT3 in Huh7 and SMMC7721 cells (Figure 6(b)).

## 4. Discussion

HCC is widely considered as the most common primary malignancy of the liver, with a high fatality rate and a great threat to human health [29]. It is a chronic hot topic to explore the effective drugs for HCC treatment. Recently, numerous anticancer compounds, such as berberine, curcumin and saponin, are found in Chinese medicines, which provides a new guidance for HCC therapy [30, 31]. In this study, we found that ZnS exhibits an inhibitory effect on malignant progression of HCC via suppressing autophagy. Furthermore, this process is associated with the inhibition of the AKR1C1-mediated JAK2/STAT3 signaling pathway.

ZnS is a saponin from the rhizome of *Dioscorea zingiberensis* C.H. Wright. Some studies have demonstrated that ZnS exhibits antiproliferative, proapoptotic, and antimetastasis effects in cancer treatment [28, 32]. Similarly, our study found that ZnS inhibited the viability and migration, as well as promoted the apoptosis of HCC cells. Besides, the levels of oxidative stress markers, including ROS, SOD, and MDA, were significantly increased in HCC cells after ZnS administration. These results indicated that ZnS exerts an anticancer effect on HCC via inhibiting cell viability and migration and promoting apoptosis and oxidative stress.

Autophagy is a conserved lysosomal catabolic process aimed at maintaining cellular homeostasis through degrading and recycling cellular components [12]. Recently, autophagy has attracted considerable attention as a potential target of drugs for cancer treatment. Our previous study has suggested that the anticancer activity of ZnS may be associated with the inhibition of autophagy [28]. Therefore, the relationship between ZnS and autophagy was further investigated. 3-MA is an autophagy inhibitor commonly used for the study of autophagy occurring in cancer. In this study, we found that 3-MA inhibited the cell viability and migration, as well as promoted the apoptosis and oxidative stress in HCC, indicating that the inhibition of autophagy can suppress the malignant progression of HCC. In addition, our study showed that ZnS suppressed the autophagosomes formation, decreased the expression of LC3II/LC3I, and increased the expression of P62 in HCC cells. These results indicated that ZnS may exert the anticancer effect on HCC by inhibiting autophagy. Furthermore, ZnS + 3-MA potentiated the inhibitory effect of ZnS on the malignant progression of HCC, suggesting a synergistic effect of ZnS and 3-MA against HCC.

AKR1C1 is an aldosterone reductase with the function of catalyzing NAPDH-mediated reduction reaction, which is highly expressed in various cancers, such as liver cancer, lung cancer, and gastric cancer [20, 33, 34]. There is overwhelming evidence that AKR1C1 exerts its anticancer effect via the phosphorylation of STAT3 and its upstream kinase JAK2 [18, 21]. Mounting researchers found that the JAK2/STAT3 pathway is involved in the anticancer process via regulating autophagy [35–37]. Our study found that ZnS treatment remarkably downregulated the expression of AKR1C1, p-JAK2, and p-STAT3 in HCC cells, whereas AKR1C1 overexpression weakened this effect. This finding indicates that ZnS can inhibit the AKR1C1-mediated JAK2/STAT3 pathway. Combined with the results of ZnS suppressing autophagy in HCC, we speculated that the anticancer effect of ZnS may be associated with the inhibition of the AKR1C1-mediated JAK2/STAT3 pathway. Moreover, we confirmed that 3-MA also suppressed the AKR1C1-mediated JAK2/STAT3 pathway and potentiated the inhibitory effect of ZnS on this pathway. Therefore, we speculate that ZnS may inhibit autophagy via moderating the AKR1C1-mediated JAK2/STAT3 signaling pathway, thereby suppressing HCC.

In conclusion, ZnS is a potential drug applied for HCC treatment, due to its inhibitory effects on the malignant progression of HCC. The anticancer effect of ZnS against HCC is closely related to the inhibition of autophagy, which may be related with the AKR1C1-mediated JAK2/STAT3 signaling pathway. This study provides a new drug for HCC treatment and offers the basis for clarifying the underlying mechanism of ZnS against HCC. However, our data are still scarce to fully elucidate the mechanism of ZnS regulating the AKR1C1-mediated JAK2/STAT3 pathway during HCC treatment. Moreover, animal and clinical trials need to be performed to verify the therapeutic effect of ZnS against HCC.

## Data Availability

The data used to support the findings of this study are available from the corresponding author upon request.

## Additional Points

ZnS inhibited the malignant progression of HCC, autophagy, and AKR1C1-mediated JAK2/STAT3 pathway. 3-MA enhanced the inhibitory effect of ZnS in HCC.
